# Supplementary material for: Comprehensive Serum Glycopeptide Spectra Analysis Combined with Artificial Intelligence (CSGSA-AI) to Diagnose Early-Stage Ovarian Cancer
Source: Cancers (Basel). 2020 Aug 21;12(9):2373. doi: 10.3390/cancers12092373 (PMC7563497; doi:10.3390/cancers12092373)
Supplement: Supplementary file 1 [file cancers-12-02373-s001.pdf]

Supplementary Material: Comprehensive Serum Glycopeptide Spectra Analysis Combined with Artificial Intelligence (CSGSA-AI) to Diagnose Early-Stage Ovarian Cancer

Kazuhiro Tanabe, Masae Ikeda, Masaru Hayashi, Koji Matsuo, Miwa Yasaka, Hiroko Machida, Masako Shida, Tomoko Katahira, Tadashi Imanishi, Takeshi Hirasawa, Kenji Sato, Hiroshi Yoshida and Mikio Mikami

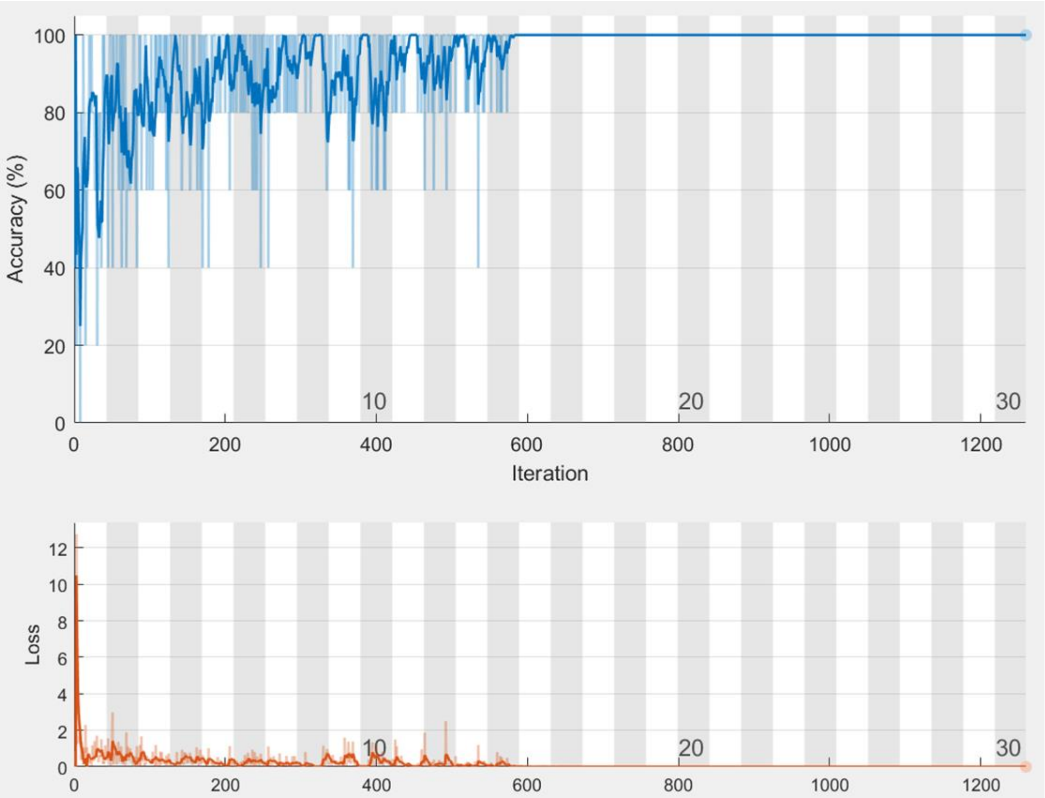

Figure 1. Accuracy and loss converging curve.

Table 1. The method of creating 2D barcodes from the levels of glycopeptide expression.

Glycopeptide Relative Intensity	Brightness
< 100	RGB (255, 255, 255)
100–300	RGB (240, 240, 240)
300–500	RGB (220, 220, 220)
500–700	RGB (200, 200, 200)
700–900	RGB (180, 180, 180)
900–1100	RGB (160, 160, 160)
1100–1300	RGB (140, 140, 140)
1300–1500	RGB (120, 120, 120)

1500–1700	RGB (100, 100, 100)
1700–1900	RGB (80, 80, 80)
1900–2100	RGB (60, 60, 60)
2100–2300	RGB (40, 40, 40)
2300–2600	RGB (20, 20, 20)
2600–3000	RGB (10, 10, 10)
> 3000	RGB (0, 0, 0)

Table 2. The method of creating 2D barcodes from the levels of glycopeptide expression and serum levels of CA125 and HE4.

Glycopeptide Relative Intensity	R	G	B
< 100	255 × CA125_factor	255 × HE4_factor	255
100–300	240 × CA125_factor	240 × HE4_factor	240
300–500	220× CA125_factor	220× HE4_factor	220
500–700	200 × CA125_factor	200 × HE4_factor	200
700–900	180 × CA125_factor	180 × HE4_factor	180
900–1100	160 × CA125_factor	160 × HE4_factor	160
1100–1300	140 × CA125_factor	140 × HE4_factor	140
1300–1500	120 × CA125_factor	120 × HE4_factor	120
1500–1700	100 × CA125_factor	100 × HE4_factor	100
1700–1900	80 × CA125_factor	80 × HE4_factor	80
1900–2100	60 × CA125_factor	60 × HE4_factor	60
2100–2300	40 × CA125_factor	40 × HE4_factor	40
2300–2600	20 × CA125_factor	20 × HE4_factor	20
2600–3000	10 × CA125_factor	10 × HE4_factor	10
> 3000	0	0	0

CA125_factor = (3 − Log10(CA125))/3.

HE4_factor = (3 − Log10(HE4))/3.

Table 3. Exclusion criteria of participants.

**Exclusion Criteria**
Affected by combination of several types of cancers
A history of hormonal drug administration due to malignant tumor, autoimmune disease and thyroid abnormality
Abnormal values in blood tests. WBC: > 9600, PLT: > 48, LDH: > 263, HB: < 9.2 and CRP > 3.0.
Renal dysfunction
Liver dysfunction
Aged 75 and above
Diagnosed as mixed carcinoma
Diagnosed as fallopian tube cancer/ peritoneal cancer
Affected massive ascites / pleural effusion
Diagnosed as endometrioma, mucinous cystoadenoma, germ cell tumor or sex cord stromal tumor
